# Memory performance on the Auditory Inference Span Test is independent of background noise type for young adults with normal hearing at high speech intelligibility

**DOI:** 10.3389/fpsyg.2014.01490

**Published:** 2014-12-22

**Authors:** Niklas Rönnberg, Mary Rudner, Thomas Lunner, Stefan Stenfelt

**Affiliations:** ^1^Technical Audiology, Department of Clinical and Experimental Medicine, Linköping UniversityLinköping, Sweden; ^2^Linnaeus Centre HEAD, Swedish Institute for Disability Research, Linköping UniversityLinköping, Sweden; ^3^Department of Behavioural Sciences and Learning, Linköping UniversityLinköping, Sweden; ^4^Oticon Research Centre EriksholmSnekkersten, Denmark

**Keywords:** speech-in-noise, cognition, working memory, updating, listening effort, cognitive spare capacity

## Abstract

Listening in noise is often perceived to be effortful. This is partly because cognitive resources are engaged in separating the target signal from background noise, leaving fewer resources for storage and processing of the content of the message in working memory. The Auditory Inference Span Test (AIST) is designed to assess listening effort by measuring the ability to maintain and process heard information. The aim of this study was to use AIST to investigate the effect of background noise types and signal-to-noise ratio (SNR) on listening effort, as a function of working memory capacity (WMC) and updating ability (UA). The AIST was administered in three types of background noise: steady-state speech-shaped noise, amplitude modulated speech-shaped noise, and unintelligible speech. Three SNRs targeting 90% speech intelligibility or better were used in each of the three noise types, giving nine different conditions. The reading span test assessed WMC, while UA was assessed with the letter memory test. Twenty young adults with normal hearing participated in the study. Results showed that AIST performance was not influenced by noise type at the same intelligibility level, but became worse with worse SNR when background noise was speech-like. Performance on AIST also decreased with increasing memory load level. Correlations between AIST performance and the cognitive measurements suggested that WMC is of more importance for listening when SNRs are worse, while UA is of more importance for listening in easier SNRs. The results indicated that in young adults with normal hearing, the effort involved in listening in noise at high intelligibility levels is independent of the noise type. However, when noise is speech-like and intelligibility decreases, listening effort increases, probably due to extra demands on cognitive resources added by the informational masking created by the speech fragments and vocal sounds in the background noise.

## INTRODUCTION

Speech understanding requires the interplay of top–down and bottom–up processes. Top–down processes include cognitive abilities that allow speech perception and comprehension (Davis and Johnsrude, 2007; Besser et al., 2013), while bottom–up processes include the perception of sound and the ability to hear. Hearing can be regarded as a mainly passive function that provides access to the auditory world via perception of sounds. Listening can then be viewed as a higher order function that requires intention and attention (Kiessling et al., 2003; Pichora-Fuller and Singh, 2006). Every day we hear many sounds, but we only listen to some of them. We hear the hum from the refrigerator but we may listen attentively to the news on the radio. Consequently, listening is required when heard information is to be processed for comprehension and to be remembered. However, the processes involved in listening, intention and attention, load on cognitive resources and therefore demand expenditure of effort (Kiessling et al., 2003; Pichora-Fuller and Singh, 2006).

In favorable listening conditions the speech signal is intact and understanding is implicit and automatic (Rönnberg, 2003; Rönnberg et al., 2008, 2013). However, when listening takes place in adverse conditions, a mismatch between the input from the speech signal and the phonological representations that are stored in long term memory may occur. Then explicit processing is needed for speech recognition. Thus, having a good cognitive capacity facilitates speech recognition in adverse listening conditions (Edwards, 2007; Akeroyd, 2008; Avivi-Reich et al., 2014). Adverse conditions may arise due to signal degradation caused by an unfamiliar speaker, competing background sounds, signal processing in a hearing aid, or hearing impairment (Stenfelt and Rönnberg, 2009; Mattys et al., 2012). Therefore, more cognitive resources appear to be needed when listening in noise than in quiet (Larsby et al., 2005; Pichora-Fuller and Singh, 2006; Edwards, 2007; Akeroyd, 2008; Mishra et al., 2013a; Ng et al., 2013a). Even though low levels of noise can be beneficial for speech perception of weak signals through stochastic resonance (Moss et al., 2004), for well audible and clear speech noise result in worse speech perception that load the cognitive resources. These cognitive resources may include working memory and executive functions (Rönnberg et al., 2010, 2013). Working memory is the ability to temporarily store and process information (Baddeley, 2000). During speech comprehension, executive functions are required to update working memory with new information and simultaneously remove old information (Miyake et al., 2000). It has been suggested that both working memory and updating processes are involved in disambiguating degraded speech and inferring absent information when listening takes place in adverse conditions (Rudner et al., 2011b). This may compensate for speech understanding difficulties (Rönnberg et al., 2008, 2013; Rudner et al., 2011a; Mishra et al., 2013a). However, it seems that the relation between speech perception in noise and working memory capacity (WMC) is stronger when speech is masked by a fluctuating masker compared to stationary noise (Gatehouse et al., 2003; George et al., 2007; Lunner and Sundewall-Thoren, 2007; Rudner et al., 2009, 2011a; Rönnberg et al., 2010; Koelewijn et al., 2012; Zekveld et al., 2013). An explanation for this might be that individuals with greater cognitive capacity are better able to utilize the short periods with increased signal-to-noise ratio (SNR) to infer information that is masked when the noise is louder (Duquesnoy, 1983), but they might also be better to inhibit the distracting effect of the noise.

Cognitive resources are consumed in the act of listening, which in turn leaves fewer resources to process the auditory information at a higher level (Rudner and Lunner, 2013). The residual cognitive resources after successful listening has taken place are referred to as cognitive spare capacity (Mishra et al., 2010; Rudner et al., 2011a). It has been shown that cognitive spare capacity is sensitive to processing load relating to both memory storage requirements (Mishra et al., 2013a,b) and background noise (Mishra et al., 2013a). Rönnberg et al. (2014) showed an effect of SNR with decreased memory performance in poorer SNR for individuals with normal hearing and high WMC, using the Auditory Inference Span Test (AIST). This test is designed to measure the ability to apply different levels of cognitive processing to auditory information as an objective measure of listening effort. These levels are designed to load differently on working memory and the executive function of updating. When background noise level increased the memory performance decreased, even though speech intelligibility levels were better than 90% (Rönnberg et al., 2014). This suggests that more cognitive resources were engaged in listening when background noise increased, which reduced residual resources needed to remember the auditory information. However, this was only true for individuals with greater WMC. This indicated that the test might be too difficult for individuals with less WMC, and that the extra demands the noise put on the cognitive system did not further decrease the overall low memory performance. Other studies have showed an effect of improved memory performance for hearing impaired individuals with high WMC when a noise reduction algorithm was used (Ng et al., 2013a). This suggests that background noise affects memory performance for individuals with normal hearing as well as individuals with hearing impairment, but that this effect is dependent on task difficulty as well as the individual’s WMC.

Limited WMC is gradually consumed by increasing processing demands when listening takes place in adverse conditions, leaving fewer resources to process and store information (Pichora-Fuller and Singh, 2006; Schneider, 2011), or in other words, leading to less cognitive spare capacity (Rudner et al., 2011a; Rudner and Lunner, 2014). Therefore, an individual with higher WMC is likely to cope better with adverse listening conditions than an individual with lower WMC (Lunner, 2003; Larsby et al., 2005; Pichora-Fuller and Singh, 2006; Foo et al., 2007; Pichora-Fuller, 2007; Rudner et al., 2009; Schneider, 2011). When a modulated masker is used, this difference is expected to be more pronounced (Koelewijn et al., 2012; Zekveld et al., 2013). Depending on the SNR, the modulated noise can divide the speech signal into intelligible and unintelligible parts. This is because the modulated noise contains short periods where the masker has low magnitude resulting in higher SNRs, where speech recognition is better, which in turn might lead to a release from masking of the target speech (Festen and Plomp, 1990). The cognitive processes, WMC and updating ability (UA), store and update unidentified disjointed parts of the speech signal, caused by the modulated masker, in working memory until the speech information can be resolved. Consequently, an individual with greater cognitive capacity is likely to be more capable to decode speech embedded in a modulated masker and thereby better speech recognition. As processing continues, the contents of working memory are continually updated with new information and old pieces of information are discarded (Rudner et al., 2011b). Therefore, an individual with greater cognitive capacity will perform better on a task that tests storage and processing of auditory information compared to an individual with fewer cognitive resources. More specifically, in easy listening conditions with low cognitive loads, there would neither be a significant performance difference between individuals with high or low WMC, nor between individuals with high or low UA, since task demands are low. However, in adverse listening conditions or when task demands require more cognitive processes, as updating information or processing of information in working memory, individuals with higher cognitive capacity are likely to perform better. Finally, when the masker is modulated, the difference in AIST performance between individuals with high cognitive capacity and individuals with low cognitive capacity is likely to be greater than in steady-state noise (Koelewijn et al., 2012; Zekveld et al., 2013).

The aim of the present study was for the first time to test whether type of noise influences listening effort measured using the AIST (Rönnberg et al., 2011) at high speech intelligibility levels. AIST performance was expected to be best in amplitude modulated noise (AMN) compared to steady state noise (SSN) and the international speech test signal (ISTS) when intelligibility was at equal level for all noise types. We also expected AIST performance to decrease with increasing noise level, as also shown by Rönnberg et al. (2014). Furthermore, we expected that participants with better cognitive capacity, i.e., higher WMC and better UA, would show better AIST performance than participants with worse cognitive capacity, similar to Rönnberg et al. (2014). Also, participants with high cognitive capacity were expected to perform better than participants with lower cognitive capacity on AIST tasks presented at poorer SNRs in modulated noise with high memory and processing demands.

## MATERIALS AND METHODS

### PARTICIPANTS

Twenty participants with normal hearing thresholds, 11 women and 9 men, with a mean age of 35 years (SD: 4.4, range 28–42) accepted to be part of the study. They were all native Swedish speakers. Baseline audiometry was done (in a sound treated room according to ISO 8253-1:2010) to verify the inclusion criteria of hearing thresholds better than or equal to 20 dB HL for the frequencies 250–4000 Hz in both ears. These frequencies were used as inclusion criteria since there is little information in the speech material used above these frequencies. Three participants did not have normal hearing for all frequencies (125–8000 Hz). One participant had a threshold of 30 dB HL at 6000 Hz at the worst ear, one participant 35 dB HL at 6000 Hz and 40 dB HL at 8000 Hz at the worse ear, and one participant 30 dB at 125 Hz at the worse ear. The participants had self-reported normal visual acuity (after correction), and no tinnitus problems. All had participated in a previous study (Rönnberg et al., 2014). The study was approved by the Regional Ethical Review Board in Linköping.

### MATERIALS

**FIGURE 1:**
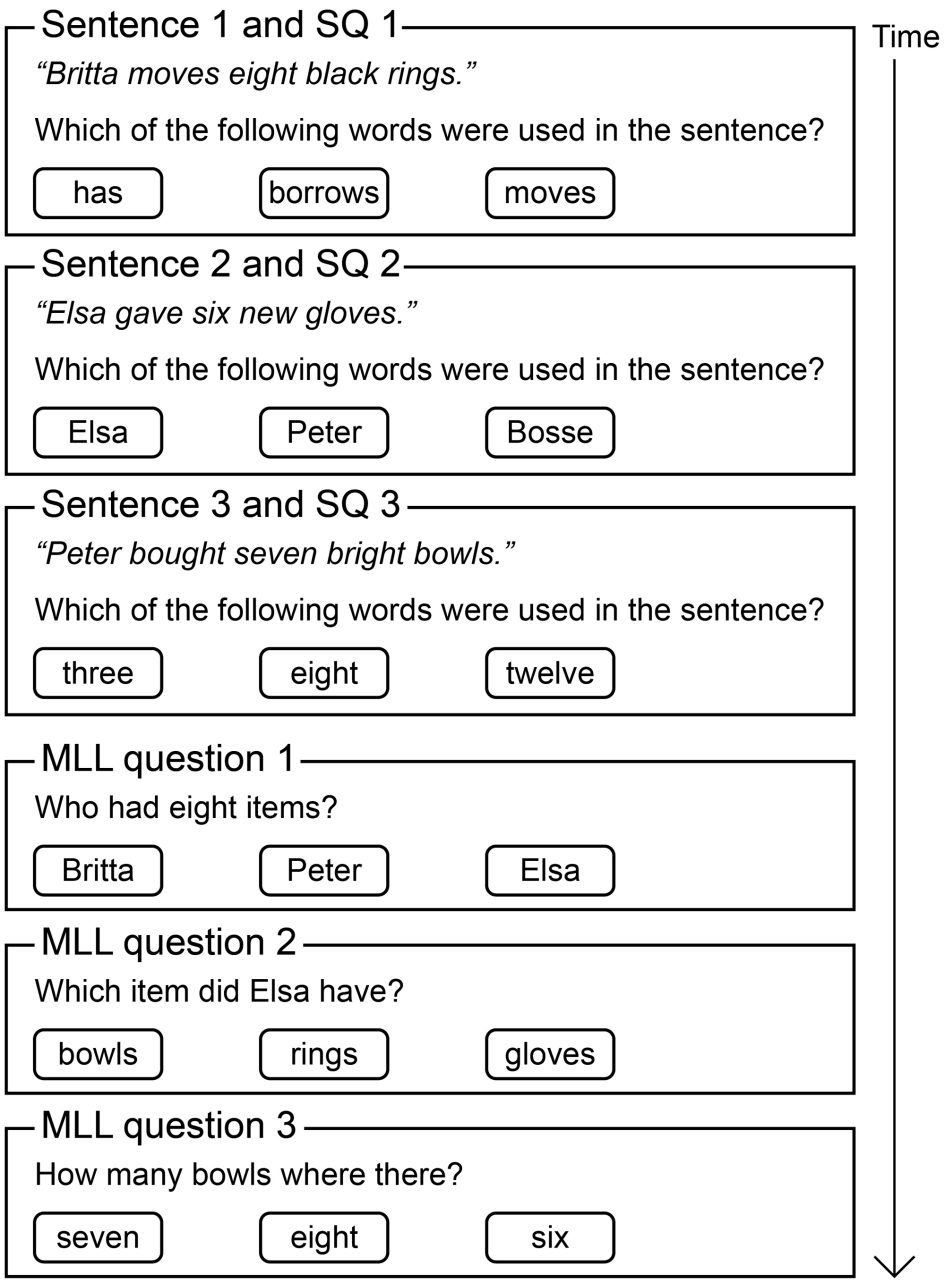
**Schematic of the Auditory Inference Span Test (AIST).** A sub-list of three Hagerman sentences with SQ are shown. These are then followed by three memory load level (MLL) questions, all of these belong to the same MLL. MLL 2 questions are shown.

The AIST test (Rönnberg et al., 2011, 2014) uses five-word matrix-type sentences in Swedish, the Hagerman sentences (Hagerman, 1982; Hagerman and Kinnefors, 1995). These sentences always have the same structure: name, verb, number, adjective, item. For example “Anna has four new gloves,” see **Figure 1**. The tokens for each category are selected from a closed set of 10 items. Thus, the Hagerman sentences have low redundancy, which makes it impossible to predict any of the words from the context provided in the sentence.

Three noise types were used in the experiment. One of these was the original speech-shaped steady state noise (SSN) by Hagerman (1982) which has the same long-term average spectrum as the speech material. The second noise type (AMN) was the same as SSN but amplitude modulated with a modulation frequency of 5 Hz and a modulation depth of 20 dB. The third noise type was the ISTS (Holube et al., 2010), which consists of six voices reading a story in six different languages. These recordings are cut into 500 ms segments, which are then randomized and concatenated. This method ensures a natural speech signal that is largely non-intelligible.

**FIGURE 2:**
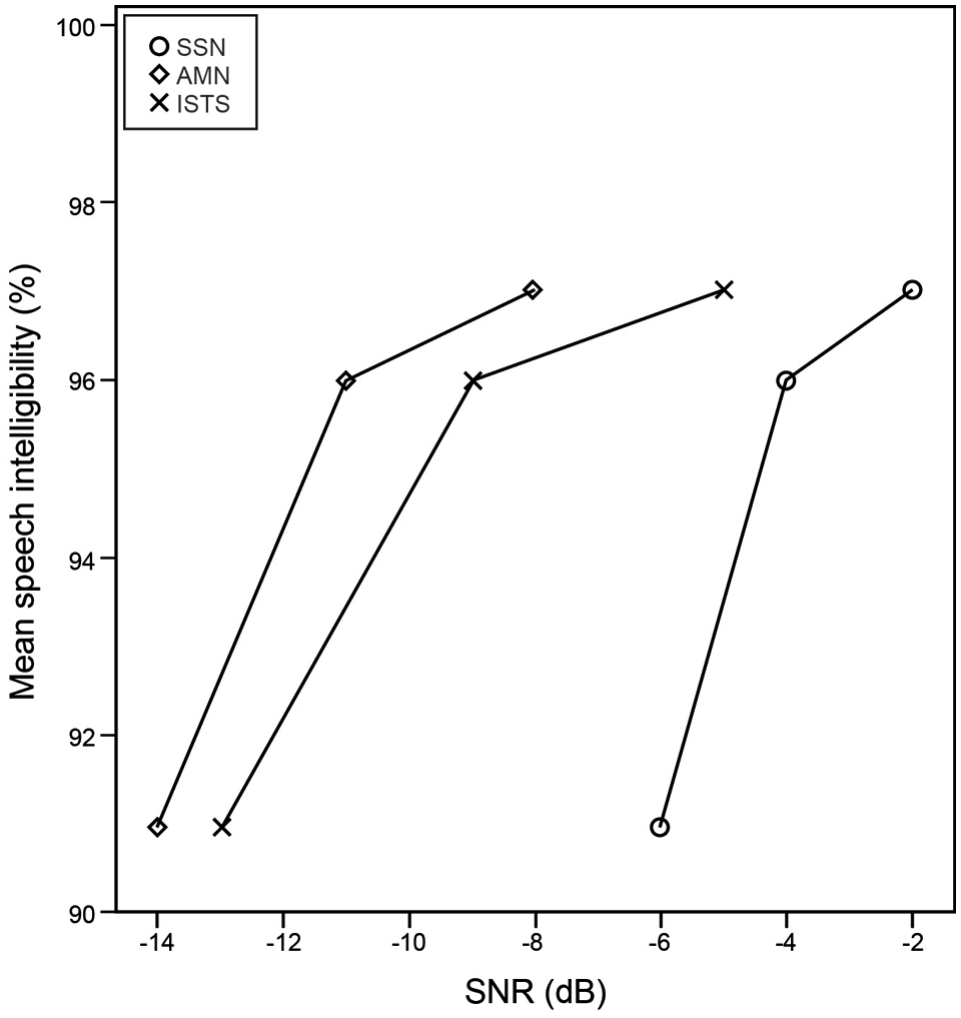
**Signal-to-noise ratio threshold for speech intelligibility levels 91, 96, and 97%**.

The test was administered at three different SNRs targeting a speech intelligibility of above 90% but below 100%, see **Figure 2**. This ensured reasonably good speech recognition, while the noise level theoretically caused a challenging listening situation. In a previous study (Rönnberg et al., 2014), the AIST was administered in SSN at three SNRs (-2, -4, and -6 dB). These SNRs corresponded to the average speech intelligibility levels of 97, 96, and 91% in SSN. Ten participants with normal hearing, none of whom took part in the present study, were recruited to determine SNRs for the same three speech intelligibility levels: 97% (SNR1), 96% (SNR2), and 91% (SNR3) for the target sentences embedded in AMN and ISTS. Matching speech intelligibility levels between noise types enabled comparison in AIST performance between noise types, and also made for a very conservative test of differences in listening effort across noise types and SNRs. The SNRs were obtained by altering the noise level, while holding the speech level constant. The sound was presented bilaterally through headphones.

### AUDITORY INFERENCE SPAN TEST

The AIST is a dual-task hearing-in-noise test, combining auditory and memory processing (Rönnberg et al., 2011). The participants’ task is to recall and process the information from the sentences and respond in a three-alternative forced-choice procedure. In the present study, a total of nine sentences, all belonging to the same original list (Hagerman, 1982) of ten sentences, were presented consecutively in each noise type at each SNR. This was to keep speech intelligibility balanced, and to avoid duplicate answer alternatives. To verify speech recognition, one word from each sentence was probed immediately after the presentation [this will be termed sentence question (SQ)]. The accuracy and timing of the responses to these questions were recorded. The AIST was administered in accordance with the standard procedure (Rönnberg et al., 2011). After each sub-list of three sentences, the participant was prompted to answer three sequentially presented multiple choice questions about the information given in the sentences, see **Figure 1**. These questions were designed to engage one of three levels of cognitive processing, called memory load levels (MLLs). Only one MLL was probed at a time, using three different questions. The multiple choice alternatives were names, numbers, or items. The order of presentation of MLLs was balanced between participants to avoid order effects. MLL 1 tapped into memory storage by asking the participant to recall which of three given words occurred in the sentences presented, e.g., “Which of the following items was used in the sentences.” This type of question could be answered simply by scanning information held in working memory. MLL 2 also tapped into memory storage but also required updating, e.g., “What item did Britta have?” This type of question could be answered by scanning the sentences to find the appropriate name, updating working memory to maintain the relevant sentence and then scanning the sentence to find the relevant item. Consequently, MLL 2 made greater demands on working memory storage and updating than MLL 1. MLL 3 was the most cognitively demanding level. It required storage and updating of information in working memory, as well as processing of the information from all three sentences presented, e.g., “Which item was there most of ?” This type of question could be answered by scanning the sentences for the relevant information and comparing between sentences to find the information that met the criterion. After that, memory could be updated to retain the appropriate sentence and identify the correct answer. Thus, MLL 3 made greater cognitive demands than MLL 2, specifically in terms of working memory storage, comparing characteristics and updating. Correct responses related equally often to the first, second, and third sentences and a balancing procedure ensured that this applied across conditions and participants. The AIST score was the number of questions that were correctly answered for each MLL in each noise type at each SNR.

### COGNITIVE TESTS

The reading span test (RS; Rönnberg et al., 1989; Daneman and Merikle, 1996) is a well-established test of working memory (Unsworth and Engle, 2007). A short version in Swedish, with a maximum score of 28, was used in the present study (Rönnberg et al., 2014). Grammatically correct three-word sentences were presented, one word at the time, on the computer screen. Half of the sentences were reasonable and half were absurd. After each sentence, the participant was asked to judge whether it made sense or not. After each set of between 2 and 5 sentences, the participant’s task was to recall in serial order either the first or the last words of each of the sentences in the set. The prompt “first” or “last” was provided only after set presentation was complete. The reading span score was the number of correctly recalled words.

The letter memory test (LM) evaluates the executive function of updating (Miyake et al., 2000). Lists of consonants were presented with capital letters one at a time on the computer screen, and the participant’s task was to recall the last four letters in the correct order. The length of the lists was either 5, 7, 9, or 11 letters long, and the presentation order was randomized. Thus, list length could not be accurately predicted. The letter memory score was the number of the four target letters that were correctly recalled in serial order for each list.

### SET UP AND TEST PROCEDURE

The AIST experiment was administered with an application developed in Matlab (R2013a; Rönnberg et al., 2014). Visual stimuli were presented on a 14^′′^ computer screen, and auditory stimuli via an M-Audio FireWire 410 audio interface through a pair of Sennheiser HDA 200 headphones with the speech level calibrated to an output level of 60 dB SPL. The testing took place in a single session in a quiet room. Even if the room was not sound attenuated, the test environment was deemed quiet enough not to affect the tests conducted. Before the test started, the participants read written instructions as a complement to instructions given orally by the test supervisor. The total testing time was at most 30 min.

### STATISTICAL ANALYSES

The data collected in this study were analyzed together with AIST performance in SSN as well as cognitive measurements of the participants collected in a previous study (Rönnberg et al., 2014). Repeated measures analyses of variance were performed on accuracy scores generated by the AIST. Bonferroni adjustment for multiple comparisons was applied as appropriate. To determine effects of other measurements on AIST performance, Pearson’s correlation analyses were used. These analyses started with total AIST score (pooled over noise type, SNR, and MLL), then AIST performance in each noise type (pooled over SNR and MLL), AIST performance in each SNR (pooled over noise type and MLL), and AIST performance in each MLL (pooled over noise type and SNR), and then AIST performance in each SNR in each noise type (pooled over MLL). All statistic calculations were performed using IBM SPSS Statistics 22.

## RESULTS

### COGNITIVE TESTS

Mean performance on the RS was 16.2 (SD = 3.7, max = 28), and mean performance on the LM was 36 (SD = 5.2, max = 48), see **Table 1**. There was no statistically significant correlation between RS and LM scores (*r* = 0.25, *p* = 0.29).

**Table 1:** Mean scores and SDs in parenthesis, for the cognitive tests and factorwise Auditory Inference Span Test (AIST) performance.

Cognitive tests				
Reading span score		16.2 (3.7), range 11–23, max 28		
Letter memory score		36.5 (5.2), range 23–46, max 48		

**AIST performance**				
**Noise type (max = 27)**	**Mean**	**95% Confidence Interval**	
		**Lower Bound**	**Upper Bound**

SSN	16.45 (4.9)	14.13	18.76
AMN	18.15 (5.1)	15.77	20.53
ISTS	16.50 (4.5)	14.41	18.59

**SNR (max = 27)**	**Mean**	**95% Confidence Interval**	
		**Lower Bound**	**Upper Bound**

SNR1	17.65 (4.2)	15.66	§19.62
SNR2	17.25 (4.4)	15.18	19.32
SNR3	16.20 (4.2)	14.16	18.24

**Noise type and SNR (max = 9)**		**Mean**	**95% Confidence Interval**	
			**Lower Bound**	**Upper Bound**

SSN	SNR1	5.40 (2.0)	4.46	6.34
	SNR2	5.75 (2.1)	4.74	6.76
	SNR3	5.30 (1.9)	4.39	6.21
AMN	SNR1	5.90 (1.8)	5.07	6.73
	SNR2	6.20 (1.9)	5.32	7.08
	SNR3	6.05 (2.1)	5.08	7.02
ISTS	SNR1	6.35 (1.7)	5.54	7.16
	SNR2	5.30 (1.8)	4.45	6.15
	SNR3	4.85 (1.7)	4.07	5.63

**MLL (max = 27)**		**Mean**	**95% Confidence Interval**	
			**Lower Bound**	**Upper Bound**

MLL 1		21.50 (3.0)	20.08	22.92
MLL 2		15.20 (5.8)	12.47	17.93
MLL 3		14.25 (5.0)	11.89	16.60

### SPEECH INTELLIGIBILITY

Speech intelligibility data collected in the previous study (Rönnberg et al., 2014) were reanalyzed in the current study. A repeated measures ANOVA with one within group variable, SNR (SNR1, SNR2, SNR3) showed a significant effect of SNR [*F*(2,38) = 27.5, *p* < 0.001, $\eta_{p}^{2}$ = 0.59]. *Post hoc* test showed a significant decrease in speech intelligibility levels between SNR1 and SNR2 (*p* = 0.035), between SNR1 and SNR3 (*p* < 0.001), as well as between SNR2 and SNR3 (*p* < 0.001). Speech intelligibility data was not collected in this study and thus speech intelligibility levels for AMN as well as for ISTS are based on the equalization data obtained from 10 subjects prior to the current study.

### AUDITORY INFERENCE SPAN TEST

**FIGURE 3:**
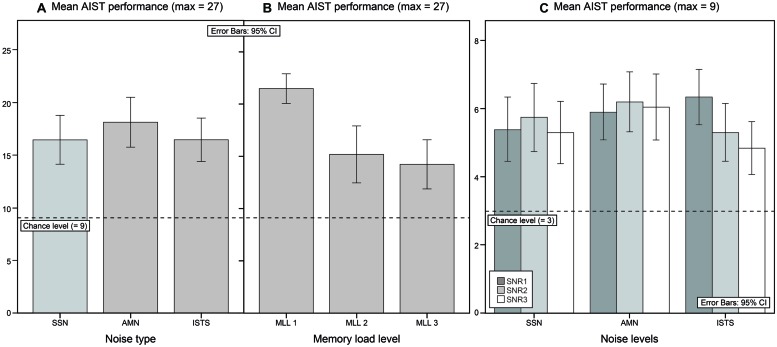
**(A)** Mean AIST performance in each noise type pooled over SNRs and MLLs. The maximum score was 27. Chance level was at 9. **(B)** Mean AIST performance for each MLL pooled over noise types and SNRs. The maximum score was 27. Chance level was at 9. **(C)** Mean AIST performance in each noise type and in each SNR pooled over MLLs. The maximum score was 9. Chance level was at 3.

The mean AIST performance in SSN was 16.4 (SD = 4.9) when performance was pooled over SNRs and MLLs (max = 27). In AMN the mean AIST performance was 18.1 (SD = 5.1), and in ISTS the mean AIST performance was 16.5 (SD = 4.5; see **Tables 1** and **2**; **Figure 3A**). The mean AIST performance in SNR1 was 17.6 (SD = 4.2), for SNR2 it was 17.2 (SD = 4.4), and for SNR3 it was 16.2 (SD = 4.4), when performance was pooled over noise types and MLLs (max = 27). The mean AIST performance was 21.5 (SD = 3.0) for MLL 1, 15.2 (SD = 5.8) for MLL 2, and 14.2 (SD = 5.0) for MLL 3, when performance was pooled over noise types and SNRs (see **Table 1**; **Figure 3B**).

**Table 2:** Mean AIST performance for each SNR in each noise type pooled over MLLs.

	SSN	AMN	ISTS	Mean
SNR1	5.40	5.90	6.35	5.88
SNR2	5.75	6.20	5.30	5.75
SNR3	5.30	6.05	4.85	5.40
Mean	5.48	6.05	5.50	

A repeated measures ANOVA with three within group variables, noise type (SSN, AMN, ISTS), SNR (SNR1, SNR2, SNR3), and MLL (MLL 1, MLL 2, MLL 3), revealed no significant effect of noise type, a tendency to significant effect of SNR [*F*(2,38) = 2.91, *p* = 0.067, $\eta_{p}^{2}$ = 0.13], and a significant effect of MLL [*F*(2,38) = 29.98, *p* < 0.001, $\eta_{p}^{2}$ = 0.61]. *Post hoc* tests showed a significant decrease in performance between MLL 1 and MLL 2 and between MLL 1 and MLL 3 (*p* < 0.001), but there was no significant difference between MLL 2 and MLL 3 (see **Table 1**; **Figure 3B**). A significant two-way interaction between noise type and SNR was found [*F*(4,76) = 2.64, *p* = 0.040, $\eta_{p}^{2}$ = 0.12; see **Tables 1** and **2**; **Figure 3C**]. Analyses of simple main effects revealed no differences in AIST performance between SNRs for SSN or for AMN, but for ISTS [*F*(2,38) = 10.01, *p* < 0.001, $\eta_{p}^{2}$ = 0.35]. *Post hoc* tests showed a significant decrease in memory performance on AIST between SNR1 and SNR2 (*p* = 0.026) as well as between SNR1 and SNR3 (*p* = 0.002), but not between SNR2 and SNR3. There were no other significant interactions.

#### AIST performance and reading span score

A significant positive correlation was found between total AIST performance and reading span score (*r* = 0.712, *p* < 0.001), showing that a higher reading span score was associated with better general AIST performance (see **Table 3**). As shown in **Table 3**, reading span score also correlated positively with AIST performance in all three noise types, in all three SNRs, as well as with all three MLLs. More specifically in SSN, reading span score correlated with AIST performance in SNR1. In the modulated noise types (AMN and ISTS), reading span score correlated with AIST performance in SNR2 as well as SNR3.

**Table 3:** The table shows correlations between total and factorwise AIST performance and cognitive measurements (WMC and UA).

Measure		WMC	UA
AIST	Total AIST	0.712**	0.319
	Total SSN	0.460*	0.199
	Total AMN	0.616**	0.210
	Total ISTS	0.623**	0.391
	Total SNR1	0.603**	0.495*
	Total SNR2	0.569**	0.237
	Total SNR3	0.715**	0.149
	Total MLL1	0.495*	0.186
	Total MLL2	0.638**	0.374
	Total MLL3	0.656**	0.214
	SSN SNR1	0.637**	0.185
	SSN SNR2	0.108	0.200
	SSN SNR3	0.391	0.093
	AMN SNR1	0.389	0.477*
	AMN SNR2	0.636**	0.118
	AMN SNR3	0.605**	0.000
	ISTS SNR1	0.340	0.512*
	ISTS SNR2	0.602**	0.218
	ISTS SNR3	0.665**	0.283

*p < 0.05, **p < 0.01.

#### AIST performance and letter memory score

Letter memory score did not significantly correlate with total AIST performance (see **Table 3**). The only significant correlation between Letter memory score and AIST performance was found between Letter memory score and AIST performance in SNR1 (*r* = 0.495, *p* < 0.05). As shown in **Table 3**, Letter memory score correlated with AIST performance in SNR1 for the modulated noise types (AMN and ISTS).

#### Sentence questions

**FIGURE 4:**
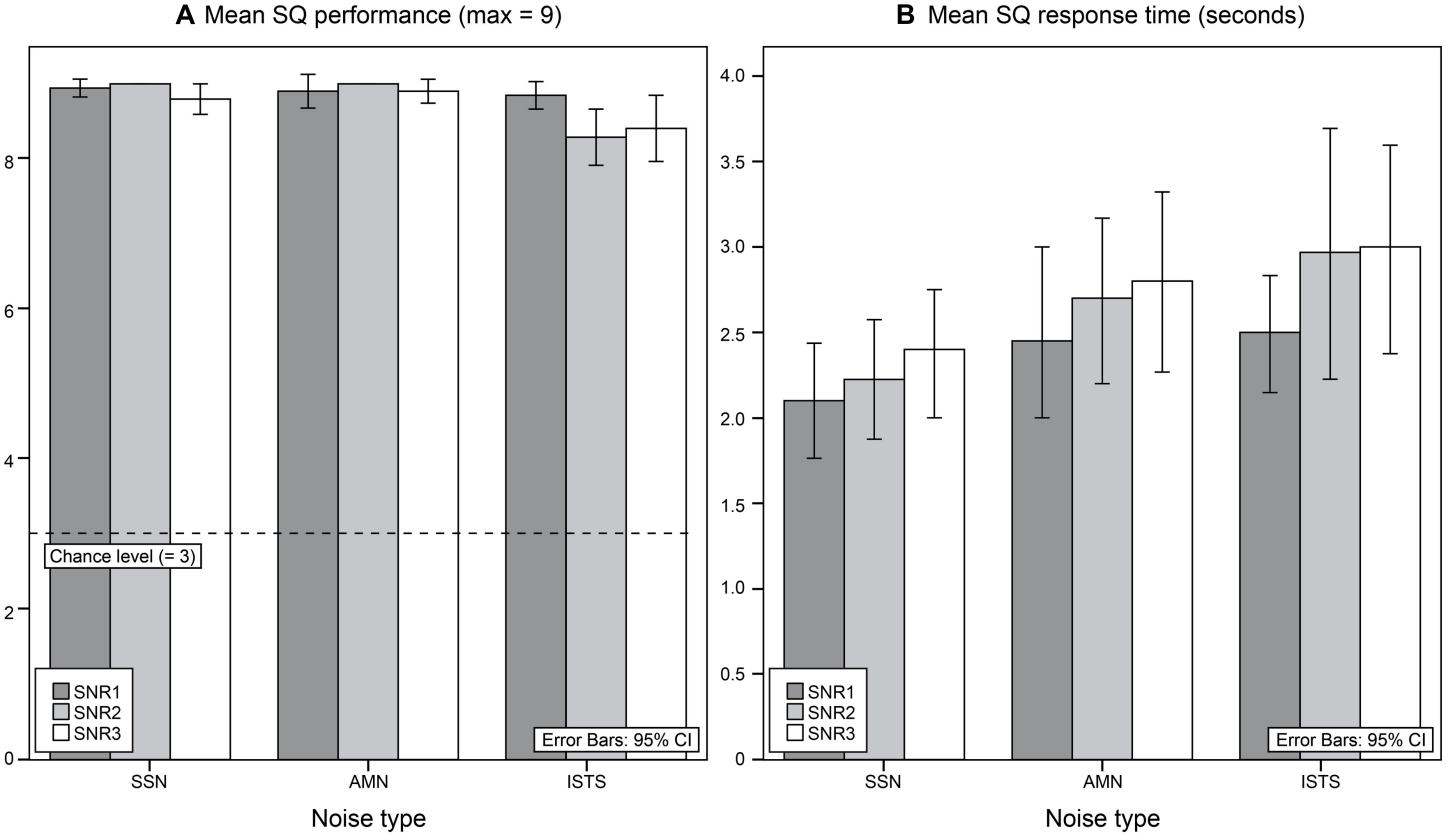
**(A)** Mean sentence question (SQ) performance for SNR type in each noise type. The maximum score was 9. Chance level was at 3. **(B)** Mean response time (in seconds) for SQ questions for each SNR in each noise type.

When SQ performance was pooled over SNRs the mean score was 26.8 (SD = 0.4) in SSN, in AMN the mean score was 26.8 (SD = 0.5), and in ISTS it was 25.7 (SD = 1.4), maximum score was 27, see **Table 4** and **Figure 4A**. A repeated measures ANOVA with two within group variables, noise type (SSN, AMN, ISTS) and SNR (SNR1, SNR2, SNR3), showed a significant effect of noise type [*F*(2,38) = 12.79, *p* < 0.001, $\eta_{p}^{2}$ = 0.40], but there was only a tendency toward significant effect of SNR [*F*(2,38) = 2.59, *p* = 0.088, $\eta_{p}^{2}$ = 0.12]. *Post hoc* tests revealed a significantly better SQ performance in SSN than in ISTS (*p* = 0.006), as well as in AMN compared to in ISTS (*p* = 0.004), but there was no significant difference in SQ performance between SSN and AMN. A significant two-way interaction between noise type and SNR was found [*F*(4,76) = 2.96, *p* = 0.025, $\eta_{p}^{2}$ = 0.14]. Analyses of simple main effects revealed significant differences in SQ performance between SNRs for ISTS [*F*(2,38) = 3.35, *p* = 0.046, $\eta_{p}^{2}$ = 0.15], but only a tendency toward significant effect for SSN [*F*(2,38) = 2.84, *p* = 0.071, $\eta_{p}^{2}$ = 0.13] and no effect for AMN. *Post hoc* tests showed a significant decrease in SQ performance in ISTS between SNR1 and SNR3 (*p* = 0.047), as well as a tendency toward significant difference between SNR1 and SNR2 (*p* = 0.074), but there was no significant difference between SNR2 and SNR3. Performance on SQs did not significantly correlate with WMC orwith UA.

**Table 4:** The table shows mean values, with standard deviations in parenthesis, for performance in each noise type in each SNR on SQ (max = 9). As well as, mean response time for each noise type in each SNR in seconds.

AIST sentence questions				
**Performance **(max = 9)****		**Mean (SD)**	**Minimum**	**Maximum**	

SSN	SNR1	8.95 (0.2)	8.00	9.00
	SNR2	9.00 (0.0)	9.00	9.00
	SNR3	8.80 (0.4)	8.00	9.00
AMN	SNR1	8.90 (0.4)	7.00	9.00
	SNR2	9.00 (0.0)	9.00	9.00
	SNR3	8.90 (0.3)	8.00	9.00
ISTS	SNR1	8.85 (0.4)	8.00	9.00
	SNR2	8.40 (0.7)	7.00	9.00
	SNR3	8.40 (0.9)	6.00	9.00

**Response time (seconds)**		**Mean**	**95% Confidence Interval**	
			**Lower Bound**	**Upper Bound**

SSN	SNR1	2.1 (0.16)	1.8	2.4
	SNR2	2.2 (0.16)	1.9	2.6
	SNR3	2.4 (0.16)	2.1	2.8
AMN	SNR1	2.4 (0.21)	2.1	2.8
	SNR2	2.7 (0.23)	2.2	3.2
	SNR3	2.8 (0.23)	2.2	3.2
ISTS	SNR1	2.5 (0.16)	2.2	2.8
	SNR2	3.0 (0.34)	2.2	3.7
	SNR3	3.0 (0.30)	2.4	3.6

When response times, see **Table 4** and **Figure 4B**, was assessed in a repeated measures ANOVA with two within group variables, noise type (SSN, AMN, ISTS), SNR (SNR1, SNR2, SNR3), a significant effect of noise type [*F*(2,38) = 5.48, *p* = 0.008, $\eta_{p}^{2}$ = 0.23] was revealed as well as a significant effect of SNR [*F*(2,38) = 5.94, *p* = 0.006, $\eta_{p}^{2}$ = 0.24]. *Post hoc* tests showed a significant increase in response time between SSN and ISTS (*p* = 0.045), but there were no significant differences between SSN and AMN, or between AMN and ISTS. *Post hoc* tests also showed a significant increase in response time between SNR1 and SNR3 (*p* = 0.010), but there were no significant differences between SNR1 and SNR2, or between SNR2 and SNR3. Response time on SQs correlated positively with WMC (*r* = 0.683, *p* = 0.001) indicating that having a greater WMC yielded in a longer response time. There was no correlation found between UA and response time on SQs.

## DISCUSSION

### SPEECH INTELLIGIBILITY

Speech intelligibility levels in SSN in the present study were identified in a larger study cohort (Rönnberg et al., 2014). The speech intelligibility levels in AMN and ISTS were matched to the speech intelligibility levels in SSN prior to the study to provide equal intelligibility levels between noise types. Even though performance on SQ is not a measure of speech intelligibility, it is nevertheless an indication of how well the participant has heard the sentence. The accuracy on SQs supported the estimated speech intelligibility levels used.

### AUDITORY INFERENCE SPAN TEST

#### Noise types

It was hypothesized that the average AIST performance would differ between noise types, even though mean speech intelligibility levels were held constant. The poorest AIST performance was expected to be found in SSN, while the best AIST performance was expected to be found in AMN. However, contrary to expectations there were no statistical significant differences in memory performance between the noise types (see **Figure 3C**). Mishra et al. (2013a) showed an increased cognitive spare capacity, as measured by improved memory performance, in ISTS compared to SSN, using lists of numbers between 13 and 99 as targets. This was not the case in the present study. The reason for this might be that the vocal sounds and speech fragments add an additional informational masking interfering more with the speech information in the sentences compared to the numbers used by Mishra et al. (2013a). This in turn would add more demands on the cognitive system leading to less cognitive spare capacity. The AMN contains short periods with less noise which might make it possible to achieve the same speech intelligibility level as for SSN but with less cognitive demands (Duquesnoy, 1983), but there was no statistical significant improved memory performance in AMN compared to SSN or ISTS (see **Figure 3C**). This suggests that for young adults with normal hearing, in SNRs targeting 90% speech intelligibility or better, the type of noise is not of importance for memory performance of the information in the sentences.

#### Signal-to-noise ratio

Speech intelligibility levels were matched between all noise types at SNR1, as well as at SNR2 and at SNR3 (see **Figure 2**). Therefore, the amount of amplitude change of the noise between SNR1 and SNR2, as well as between SNR2 and SNR3, differed between noise types, i.e., SNR1 was different in different noise types but corresponded to the same speech intelligibility level (see **Figure 2**). Access to the information in the sentences is essential for accurate AIST performance. Since all SNRs gave a mean speech intelligibility level of 90% or better, access to the information was not appreciably limited at any of the SNRs (see **Figure 2**).

Based on the previous study (Rönnberg et al., 2014), we hypothesized that a decreased SNR would force an increase in cognitive processing of auditory information, leading to less cognitive spare capacity resulting in reduced AIST performance. The tendency toward a statistically significant effect of SNR on AIST performance (see **Tables 1** and **2**; **Figure 3C**) suggested that the cognitive spare capacity, as measured by memory performance on AIST, was reduced by increasing noise level. Similar results have also been found in other studies (Mishra et al., 2013a; Ng et al., 2013a,b; Rönnberg et al., 2014). However, in the present study, increasing noise level only reduced AIST performance when ISTS was used as background noise. This suggests that increasing background noise at the high intelligibility levels used in the present study only influences listening effort when noise is speech-like (see **Figure 3C**).

When listening in AMN, young adults with normal hearing are likely to be able to utilize the short periods with increased SNR to infer information that is masked when the noise level is louder (Duquesnoy, 1983) which would give rise to release from masking (Festen and Plomp, 1990). As a result, the decrease in SNR for AMN might not be particularly more demanding when listening in SNRs targeting 90% speech intelligibility or better. Nevertheless, for ISTS, the noise level seemed to have an impact on the cognitive processes involved leading to less cognitive spare capacity and decreased memory performance on AIST (see **Tables 1** and **2**; **Figure 3C**). Even if the ISTS is largely non-intelligible (Holube et al., 2010), the voices and speech fragments in ISTS may promote informational masking (Francart et al., 2011) which would add to the cognitive load since ISTS will interfere with the Hagerman sentences at different linguistic levels (Tun et al., 2002; Brouwer et al., 2012). Consequently, since ISTS adds more cognitive load, AIST performance in ISTS is more sensitive to decreased SNR than in the other noise types. As a result, the decrease in AIST performance with worse SNR in ISTS cannot be explained by reduced intelligibility alone since SNR did not significantly affect AIST performance in SSN or in AMN.

Interestingly, the correlations with WMC, i.e., reading span score, indicated that WMC had an impact on performance in AIST when presentation took place in SSN with SNR1, but not with the other SNRs (see **Table 3**). A reason for this might be that SSN masks the signal at worse SNRs, and when the signal becomes inaudible, a greater WMC does not improve speech intelligibility. On the other hand, when SNR is better and the signal is only partly masked by the SSN, a greater WMC might facilitate speech intelligibility by storing partly heard sounds of the speech signal until these can be disambiguated. The relation between speech recognition in noise and WMC is more evident in modulated noise where individuals with high WMC have better speech recognition in noise performance compared to individuals with less WMC (Gatehouse et al., 2003; George et al., 2007; Zekveld et al., 2013), which might also explain the relation between WMC and AIST performance in SSN. For the modulated noise, WMC was of importance for memory performance when the SNR was more demanding (see **Table 3**). This suggests that when listening takes place in more troublesome listening conditions, such as increased SNR and modulated noise, WMC is more occupied with listening, and individuals with higher cognitive capacity are likely to have more cognitive spare capacity after listening and consequently perform better on the memory task than individuals with less cognitive capacity. Consequently, individuals with greater cognitive capacity will probably experience less listening effort than individuals with less cognitive capacity. On the other hand, when listening takes place in modulated noise in SNR1, the listening condition might be described as fairly simple which explains why, the extra WMC capacity did not add an additional advantage.

Another way to explain the correlations between AIST performance and WMC is based on attention. One may expect that a person with a higher WMC is better to filter out the desired signal (speech) and suppress the unwanted signal (noise) than a person with worse WMC. There are indications of such mechanisms in the literature. In an auditory brainstem response measurement it was found that the neural amplitude increased when focusing on the signal and decreased when adding a cognitive load (distractor; Sorqvist et al., 2012). This modulation of the neural response was correlated with the persons WMC. Other studies have indicated that attention and WMC correlates with spatial speech recognition performance in adults (Neher et al., 2011) and that attention supports language processing in children (Astheimer et al., 2014). However, there are other studies that have found correlation between WMC and speech perception that is unrelated to attention skills (Tamati et al., 2013). The current study did not measure attention *per se*, but it is very plausible that a better WMC facilitated auditory attentional filtering of the sentence and thereby improved both speech recognition and ability to store the information crucial for AIST performance.

Updating ability, i.e., Letter memory score, did not correlate with total AIST performance (see **Table 3**). However, having a greater UA improved AIST performance in SNR1, more specifically for SNR1 in the modulated noise types (AMN and ISTS) but not in SSN. This is consistent with the previous study where no interactions were found between AIST performance and SNRs when UA was used as a between-group variable and SSN was used as masker (Rönnberg et al., 2014). In the modulated noise types, at the best SNR, listening is fairly undemanding why having a higher UA facilitates performance on AIST. However, when the SNR gets worse, there was no effect of UA on AIST performance. Nevertheless, there was an effect of WMC on AIST performance in worse SNRs suggesting that in more troublesome listening conditions WMC is of more importance for listening than UA. WMC improves memory performance in SSN in the easiest SNR, but UA does not improve memory performance. However, in modulated noise, WMC facilitates memory performance in the worst SNR, while UA improves memory performance in the best SNR.

#### Memory load level

Auditory Inference Span Test accuracy was, as expected, a function of MLL (see **Table 1**; **Figure 3B**), where performance decreased with increasing level of memory load (Mishra et al., 2013a,b; Rönnberg et al., 2014). As in the previous study (Rönnberg et al., 2014), there were no significant difference in performance on MLL2 and MLL3. Even though performance at MLL2 and MLL3 is low, performance on both MLLs are clearly above chance level. The results suggested that regardless of MLL, WMC improves memory performance on AIST. A similar effect was found in a previous study (Rönnberg et al., 2014). Also, in the previous study (Rönnberg et al., 2014) an interaction between MLL and UA showed a benefit of high UA on questions demanding more updating of information, i.e., MLL 2. This relation was not found to be significant in the present study (see **Table 3**).

#### Response time

Response times on MLL questions were registered in the AIST process. These response times on MLL questions were not included in the analyses. The reason for this was that the measure of response time started when the question was presented on the computer screen and continued until an answer had been given, and the test had continued to the next question. Consequently, the time it took to read and comprehend the question was part of the measured response time. However, there is a difference in the complexity of the questions, why differences in response time might be due to differences in the amount of time it took to read and comprehend the question. Nevertheless, response times on MLL questions might be analyzed when pooled over the three MLLs. It was expected that response times then would be dependent on SNRs and noise types. However, no statistically significant effect of SNR or of noise type was not found. Pooled response times on MLL questions did not change with listening conditions. Consequently, response time on AIST was not deemed to be a useful measure.

### SENTENCE QUESTIONS

Performance on SQs decreased in ISTS compared to SSN and AMN, and there was an effect of SNR in ISTS but not in SSN or AMN, see **Figure 4A**. Since SQ might be considered a measure of speech recognition in the sense that the question probes that the sentence was heard, even if the three-choice procedure facilitates performance by giving possible answer alternatives as well as having a chance level of 33%, the results suggested that the general speech intelligibility levels were at the expected levels above 91% (Rönnberg et al., 2014). However, the effect of SNR only found in ISTS might suggest that speech intelligibility levels were not perfectly matched between noise types. Nevertheless, the results might also imply that speech-shaped noise in these rather favorable SNRs did not load the cognitive system to such a degree as the vocal sounds and speech fragments in ISTS did, and consequently there was no effect of SNRs for SSN and AMN on SQ accuracy. Even if ISTS is largely non-intelligible (Holube et al., 2010), it may cause additional informational masking (Francart et al., 2011) and consequently add to the cognitive load since the masker interferes with the speech material at different linguistic levels (Tun et al., 2002; Brouwer et al., 2012).

The analyses of SQ response times were based on response times correct answers as well as for incorrect answers, as there was no statistically significant difference in response time between correct and incorrect answers. Response time on SQs was an effect of noise type, with longer response times in ISTS compared to SSN and AMN. There was also an effect of SNR with increasing response times in SNR3 compared to SNR1, see **Figure 4B**. The results suggest that more processing was needed in the more problematic listening conditions (in ISTS compared to SSN, and in SNR3 compared to SNR1) and that this processing takes longer, with longer response times as a result. It seems likely to assume that the longer response time is a measure of listening effort. SQ response time correlated with WMC and not with UA. Contrary to expectations that having a greater WMC would imply faster access time to information stored in working memory and a shorter time to retrieve the position of the correct answer alternative, instead the results showed that greater WMC rather meant longer response times. The results suggested that individuals with greater WMC spent more time reading the answer alternatives and pondering the answer; however, they did not gain from this extra time spent when considering accuracy on SQ questions. Also, having a higher WMC implies having more information held in working memory, resulting in more information to scan which would require a longer time to find the matching answer.

### THE COGNITIVE MEASUREMENTS

Both the RS and the LM are delivered in visual modality, unlike the AIST which is delivered in auditory modality with visually presented multiple choice responses. This is a strength of the study, since the measurements of WMC and of UA are independent on the individual’s hearing status. Furthermore, the AIST is intended to be used in the hearing aid fitting process to assess listening effort, then it is of even greater importance that the measurement of the individual’s cognitive capacity is unaffected by the hearing status.

### CLINICAL IMPLICATION

Performance on AIST can be expected to be lower for individuals with hearing impairment than for individuals with normal hearing. A hearing impairment decreases the signal fidelity (Plomp, 1978; Pichora-Fuller and Singh, 2006), which in turn increases the cognitive involvement in listening and consequently leaves less cognitive capacity for memory storage (Rudner et al., 2011b; Picou et al., 2013) which would be measurable with the AIST. It is well established that successful hearing aid fitting needs to take individual differences in cognitive capacity into account (Lunner et al., 2009). Hitherto, cognitive measures such as reading span have been used to demonstrate associations with ability to repeat and recall speech. The advantage of a test such as AIST is that it has the potential to measure the listening effort expended by the individual under different sets of listening conditions in which noise types, SNR and potentially hearing aid settings can be manipulated. This will allow better hearing aid fitting in the future and provides an important tool for the development of better hearingaids.

## CONCLUSION

The results suggest that for young adults with normal hearing the cognitive spare capacity is reduced when background noise consists of voices and the SNR decreases. However, when speech intelligibility levels are kept constant, different masker types do not have different effects on cognitive spare capacity, at least not for intelligibility levels above 90%.

## Conflict of Interest Statement

The authors declare that the research was conducted in the absence of any commercial or financial relationships that could be construed as a potential conflict of interest.
